# Novel variants underlying autosomal recessive intellectual disability in Pakistani consanguineous families

**DOI:** 10.1186/s12881-020-00998-z

**Published:** 2020-03-24

**Authors:** Muhammad Ilyas, Stephanie Efthymiou, Vincenzo Salpietro, Nuzhat Noureen, Faisal Zafar, Sobiah Rauf, Asif Mir, Henry Houlden

**Affiliations:** 1 Department of Biological Sciences, International Islamic University Islamabad, Islamabad, 44000 Pakistan; 2 Department of Neuromuscular Disorders, UCL Institute of Neurology, Queen Square, London, WC1N 3BG UK; 3 Department of Pediatric Neurology, Children’s Hospital and Institute of Child Health, Multan, 60000 Pakistan; 4 National Center for Bioinformatics, Quaid-i-Azam University, Islamabad, Pakistan

**Keywords:** Intellectual disability, *VPS53* gene, *GLB1* gene, *MLC1* gene, Whole exome sequencing

## Abstract

**Background:**

Intellectual disability (ID) is both a clinically diverse and genetically heterogeneous group of disorder, with an onset of cognitive impairment before the age of 18 years. ID is characterized by significant limitations in intellectual functioning and adaptive behaviour. The identification of genetic variants causing ID and neurodevelopmental disorders using whole-exome sequencing (WES) has proven to be successful. So far more than 1222 primary and 1127 candidate genes are associated with ID.

**Methods:**

To determine pathogenic variants causative of ID in three unrelated consanguineous Pakistani families, we used a combination of WES, homozygosity-by-descent mapping, de-deoxy sequencing and bioinformatics analysis.

**Results:**

Rare pathogenic single nucleotide variants identified by WES which passed our filtering strategy were confirmed by traditional Sanger sequencing and segregation analysis. Novel and deleterious variants in *VPS53*, *GLB1*, and *MLC1*, genes previously associated with variable neurodevelopmental anomalies, were found to segregate with the disease in the three families.

**Conclusions:**

This study expands our knowledge on the molecular basis of ID as well as the clinical heterogeneity associated to different rare genetic causes of neurodevelopmental disorders. This genetic study could also provide additional knowledge to help genetic assessment as well as clinical and social management of ID in Pakistani families.

## Background

Next generation sequencing (NGS) methods have diversified the field of medical genetics. The increase in the number of causative ID genes is directly associated with the implementation of NGS technology for the diagnosis of patients [1]. Intellectual disability (ID) is often part of a wide spectrum of neurodevelopmental disorders, with a total prevalence of 1–3%, around the globe [2]. ID is characterized clinically by below average intellectual functioning of the human brain and adaptive behaviours which occur before the age of 18 years [3]. Whole exome sequencing (WES) is highly useful to identify rare genetic variants implicated in ID. Till 2016, only 746 genes were directly associated with ID based on clinical features and cognitive assessments, with more than 50% of these genes causing autosomal recessive ID [4]. The list of known and candidate ID genes has been increasing rapidly in the last few years according to the sysID database, with at least 1222 primary 1127 candidate genes [4].

Clinical and molecular diagnosis of ID is challenging due to its phenotypic and molecular heterogeneity. WES-based studies investigating patients with ID of variable severity, have shown a low yield of causative variants ranging from 16 to 68% [5–10] due to this heterogeneity. The identification of the genetic etiology underlying autosomal dominant ID still remains elusive in many cases. The low yield of diagnosis in this group may be due to its syndromic nature, the reduced penetrance, the lack of availability of genomic material from additional family members for segregation studies, and the yielding of a large number of variants potentially contributing or causing the patients’ phenotypes.

In contrast, the diagnosis yield of ID-causing genes in consanguineous families is often higher [11]. In literature, cohorts consisting of consanguineous families from the Greater Middle East including Pakistan, Iran and Saudi Arabia show a diagnostic yield up to 90% in several WGS/WES studies [9, 12–16].

In our study, we identified three novel homozygous variants in different ID-related genes. These results were obtained by carrying out WES in consanguineous Pakistani families. The involvement of novel variants in autosomal recessive inheritance is supported by linkage analysis by using short tandem repeat, homozygosity-by-descent (HBD) mapping, brain expression databases and published literature related to neurodevelopmental disorders.

## Methods

### Patients

The study was approved by the board of advanced studies research and ethical committees of the International Islamic University, in Islamabad, Pakistan, and in University College London, UCL Institute of Neurology according to the declaration of Helsinki. The study included five probands originating from south Punjab in Pakistan. Blood samples was collected from all family members and genomic DNA extraction was performed by the phenol chloroform extraction method. Linkage analysis was performed with STR markers mapping genes causing autosomal recessive ID. STR markers were obtained from the LDB genetic map database of the Psychiatric University Hospital in Zurich, Switzerland. However, the genotypes of families remained unclear due to uninformative microsatellite markers and therefore patient samples underwent Whole Exome Sequencing (WES) analysis.

### Whole exome sequencing and analysis

WES was performed on the probands selected from subjected families. WES was carried out on an Illumina platform HiSeq 2500 systems on average coverage of 150× by Macrogen Company (Geumcheon-gu, Seoul, South Korea). To filter the patients fastQ files, quality control tool such as Trimmomatic [17] applied to generate clean reads. Then reads were aligned to the reference human genome (GRCh38) using the Burrows Wheeler Aligner (BWA) tool and duplicate removed using Picard. The variant calling process was performed using the Genome analysis tool kit (GATK). Initially common and intronic variants were removed. All functional variants were prioritized for rare variants by filtering through databases [18] such as Exome Aggregation Consortium (ExAC) [19]. Only homozygous or compound heterozygous, non-synonymous, frameshift, splice site and coding indel variants with allelic frequencies of less than < 0.001% in the 1000 genome project and ExAC database were selected for further analysis. The variants with allelic frequencies of < 0.001% were shortlisted and pathogenic scores were checked by using Ensemble genome browser.

### Sanger sequencing

Sanger sequencing primers were designed on Primer 3 plus (www.primer3plus.com) for the shortlisted variants from all three families. PCR products were amplified using allele specific primers for selected variants (see Additional File 1, Table 1). Sequencing analysis was performed using an ABI-3730 DNA analyzer.

### Bioinformatics analysis

Models carrying the variants were constructed for each family in order to check the effect of the variant on a normal protein model by using the corresponding swiss model. Multiple alignments were performed across orthologous classes of different species and showing absolute conservation in all three families.

## Results

### Family MR-4

#### Clinical details

**Fig. 1:**
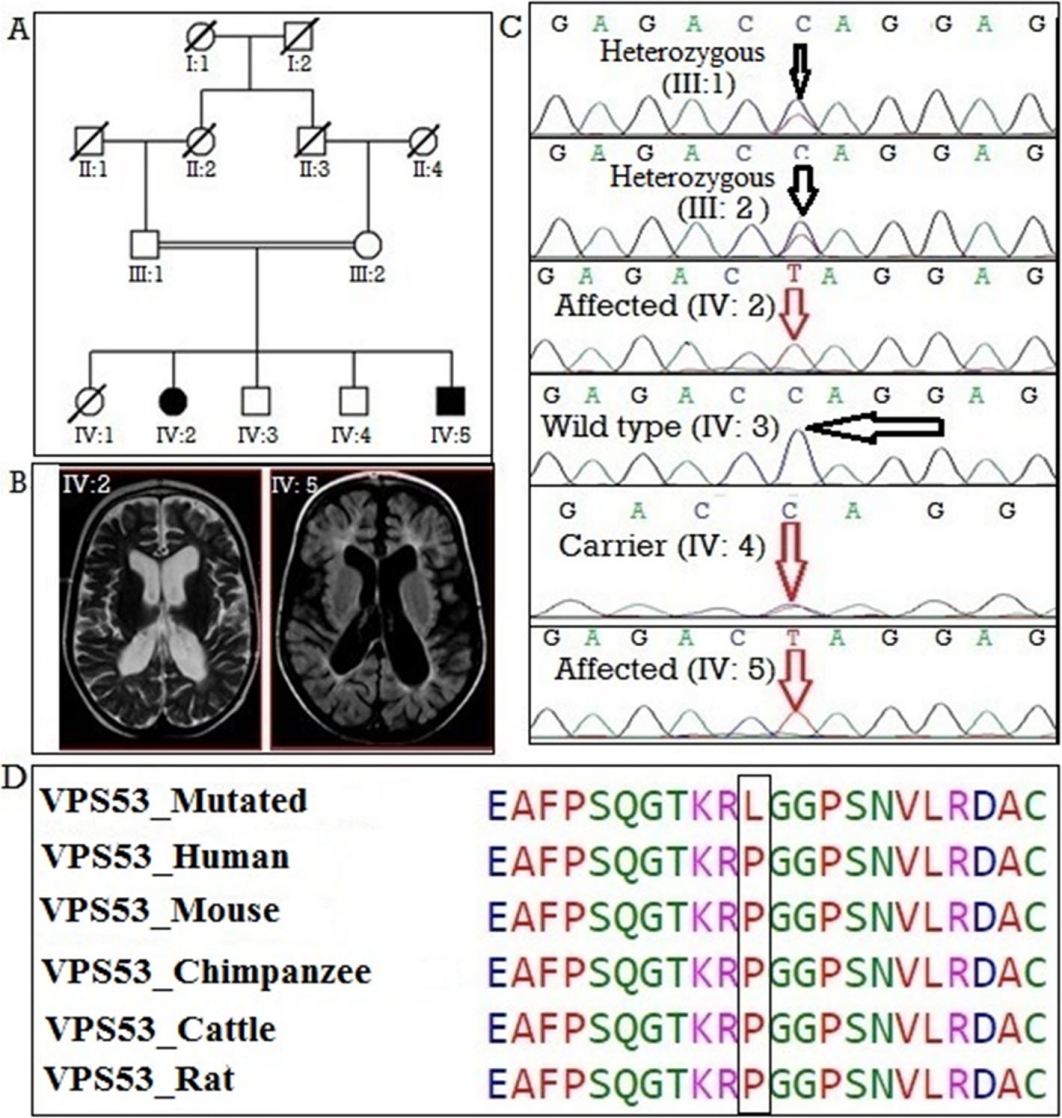
**a** Pedigree showing segregation of *VPS53* in an autosomal recessive pattern. Square and circle represent males and females, respectively. All filled circles and squares are showing affected members. **b** Brain MRI of (IV: 2) showing subtle hypodensity in gray matter more marked in left basal ganglia and patient (IV: 5) representing cerebellar atrophic changes, giant cisterna magna (indication of cerebellar hypoplasia and growth retardation of the brain). **c** Chromatograms of the respective parts of *VPS53* indicating the carrier parents (III: 1, III: 2), wild-type allele in a normal child (IV: 3), homozygous missense pathogenic variant c. C605T, (p.Pro202Leu), in an affected member (IV: 2 and IV: 5). **d** Multiple alignment representing complete conservation of the mutated amino acid (p.Pro202Leu) across different species

Two patients from the consanguineous Pakistani family MR-4 were studied (IV: 2 and IV: 5 Fig. 1a). Both pregnancy and birth were normal in both affected individuals. Head circumference was normal at birth. In the first proband (IV: 2), cognitive development was initially normal, but irascible behaviour was noticed in the early months of life. After 8 to 10 months, psychomotor delay became evident with reduced head size. The proband’s age of onset of generalized tonic-clonic seizures was 3 years and developed maximum one seizure per day which lasted up to 5 minutes. She has progressive spasticity accompanied by microcephaly, failed to achieve -4SD at the age of 3 years. Brain CT scan performed at 3 years of age demonstrated subtle hypodensity in gray matter more marked in the left basal ganglia and cerebral atrophic changes (Fig. 1b). The second proband (IV: 5) carried similar phenotypes as described above. His age of onset of seizures was 5 years. He developed maximum 10 seizures per day which lasted up to 1 min. Brain CT scan demonstrated cerebellar atrophic changes with subcortical cysts (Fig. 1b). Electroencephalogram test results demonstrated hypsarrhythmia (specific EEG pattern seen in both patients with structural abnormalities of the brain). Metabolic testing done in both patients was all within normal limits.

#### Genetic study

WES performed in family MR-4 patients (IV: 2 and IV: 4) (Supplementary Table 2) revealed a homozygous missense variant in *VPS53* shared by the 2 siblings (c.C605T, p.Pro202Leu) (Additional File 1, Table 2). *VPS53* gene is located on chromosome 17 and consists of 18 exons. This variant in *VPS53* (OMIM 615851) is disease causing based on the variant’s pathogenic score (Table 1). The variant was validated in this family through co- segregating analysis. Parents (III: 1 and III: 2) were heterozygous carriers, while both affected (IV: 2 and IV: 5) were homozygous and the unaffected sibling (IV: 4) was wild type (Fig. 1c). Multiple species alignment was performed which showed complete conservation of the affected amino acid residue (p.Pro231Leu) across different species (Fig. 1d).

**Table 1:** Variants identified in the families with ID and neurodevelopmental disorders

Gene	cDNA	Protein	Mutation Taster	Provean	CADD Score	GERP_RS
*VPS53*	c.C605T	p.P202L	Disease_causing	Deleterious	10	3.83
*GLB1*	c.C1318T	p.H440Y	Disease_causing	Deleterious	30	5.35
*MLC1*	c.C959A	p.T320K	Disease_causing	Deleterious	29.9	3.9

### Family MR-7

#### Clinical details

**Fig. 2:**
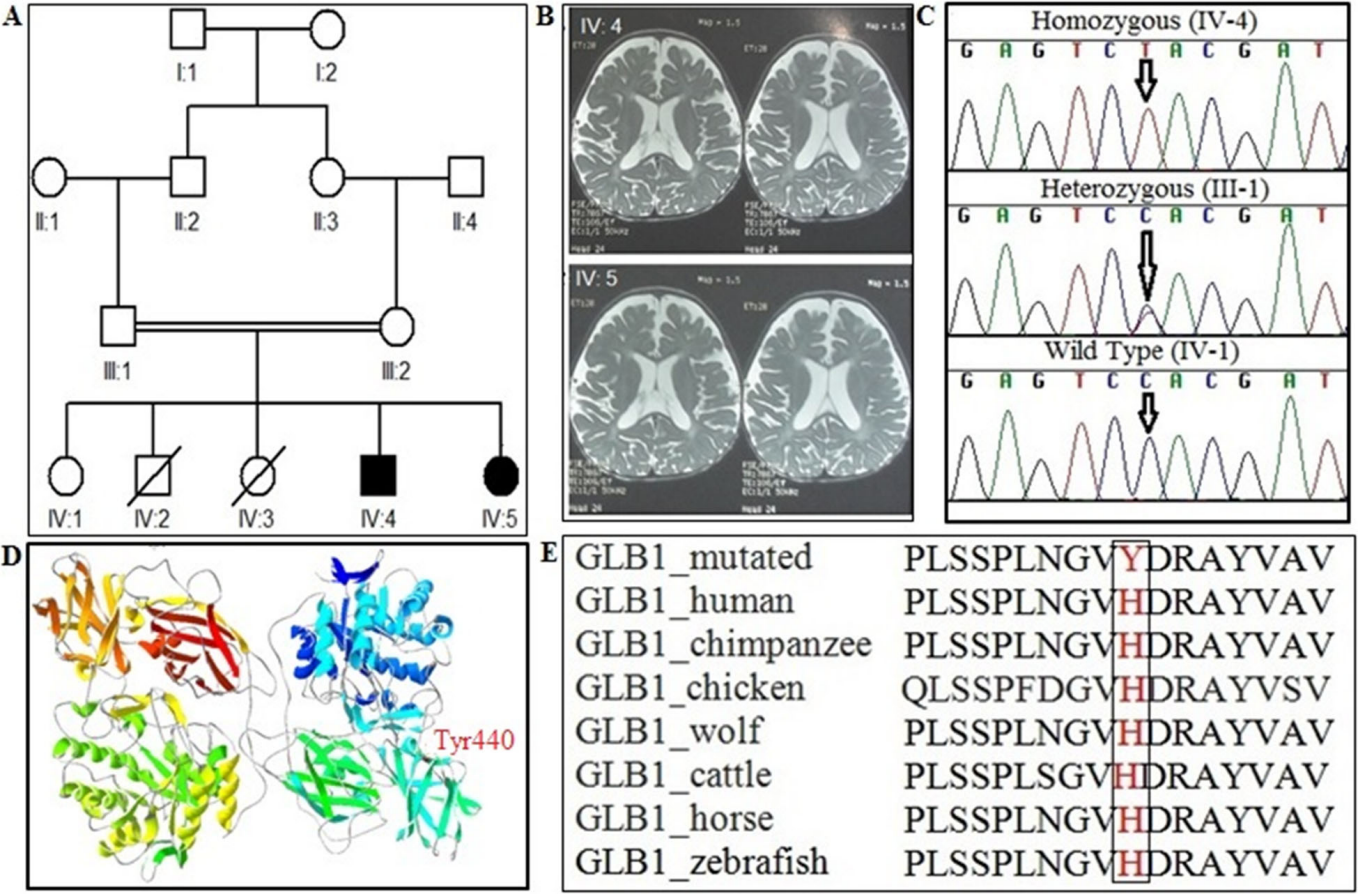
**a** Pedigree of the family MR-7 showing pattern of autosomal recessive inheritance. Clear circles and squares symbols represent normal and filled circles and squares showing affected individuals. **b** Brain MRI of T1-weighted image of patient (IV: 4) showing abnormal deep white matter signal in subcortical, prominent ventricular and extra vent spaces. Brain MRI of patient (IV: 5) showing cerebellar emotional changes with reduced periventricular deep white matter. **c** Sequence chromatograms of the *GLB1* gene indicating the homozygous mutation (c.C1318T: p.His440Tyr) in affected patients (IV: 4), wild type (IV: 1) and heterozygous carrier parents (III: 1). **d** Protein model of the gene was showing the mutated protein (Tyr440). **e** Multiple alignment of the *GLB1* gene across different orthologous species were showing complete conservation at the site of mutation

In family MR-7, two patients (IV: 4 and IV: 5 Fig. 2a) were the 4th and 5th children of healthy, consanguineous parents from Pakistan. A 3 year old boy (IV: 4) manifested developmental regression and seizures at the age of 1.5 years. His disease progressed slowly, and gradually he lost his motor skills and became bed ridden and unable to sit without support. His head occipital frontal circumference size is 45.5 cm, considered as microcephalic, and he is not able to talk. Brain MRI scans demonstrated abnormal deep white mater signal in subcortical area, prominent ventricular and extra vent spaces. EEG results show diffuse slowing of background activity (Fig. 2b). The second proband (IV: 5) presents with developmental regression which started at the age of 9 months. She is able to talk just a few words, and her head circumference is overly large. Brain MRI scans demonstrated cerebellar emotional changes with reduced periventricular deep white matter (Fig. 2b).

#### Genetic study

The two patients (IV: 4 and IV: 5) were subjected to WES to identify the underlying genetic causes of the disease (Additional File 1, Table 3). Genomic Evolutionary Rate Profiling (GERP) and Combined Annotation Dependent Depletion (CADD) Phred pathogenic scores were also analyzed by using the Ensembl variant effect predictor (VEP) tool. WES data and all online pathogenic score predictors pinpoint that variant c.C1318T, p.His440Tyr in the *GLB1* (OMIM: 622458) gene is the most probable cause of the phenotype of family MR-7 (Table 1). Co-segregation analysis showed complete segregation of the *GLB1* variant in all family members (Fig. 2c). Swiss modeling was used to generate the normal and muted protein structure of the *GLB1* gene shown in (Fig. 2d). Multiple alignment were performed for the *GLB1* gene across orthologous species showing complete conservation in the region of *GLB1* gene (Fig. 2e).

### Family MR-8

#### Clinical details

**Fig. 3:**
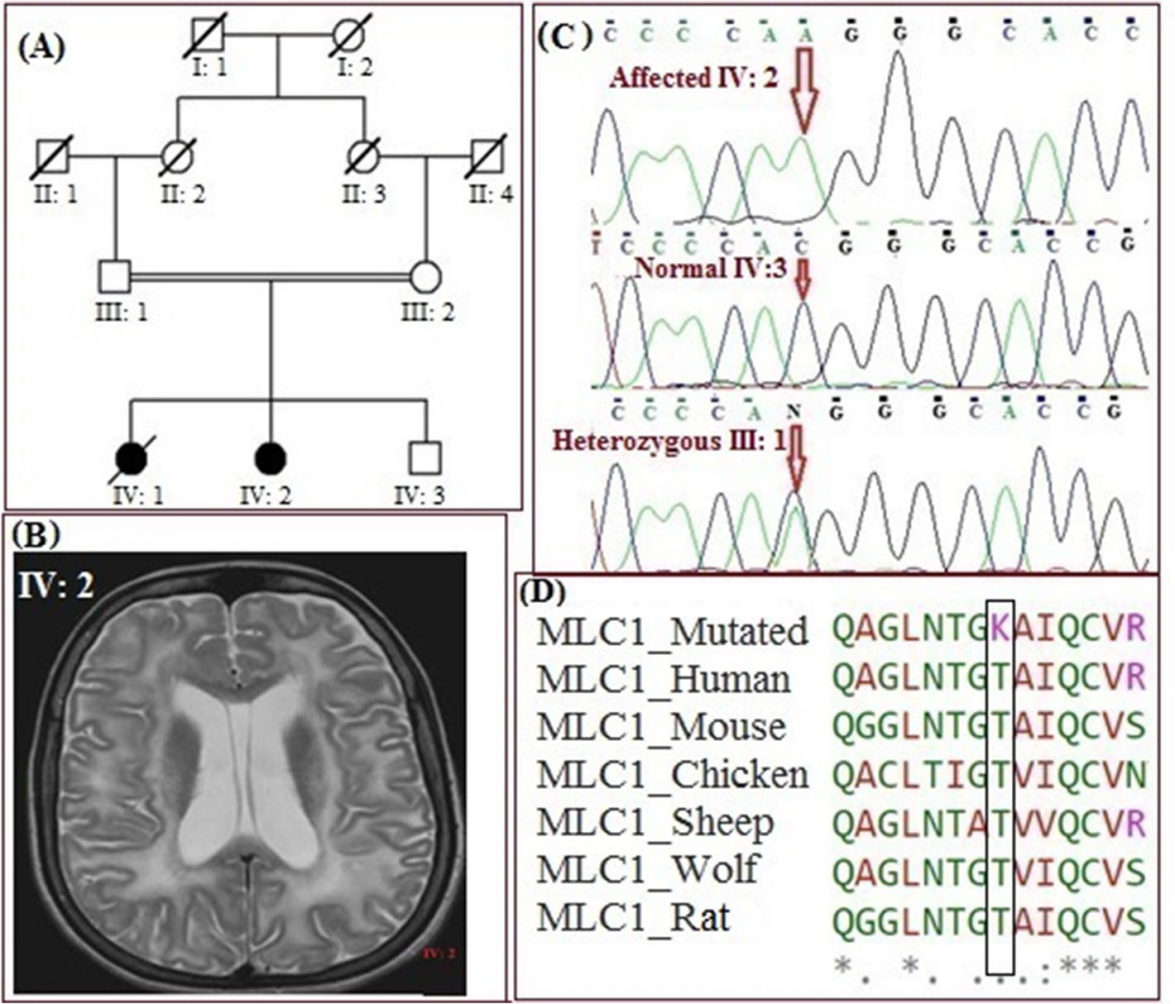
**a** Pedigree of the family representing one patient (IV: 2). **b** Brain MRI of patient showing white matter with subcortical cysts. **c** Sanger sequencing showing homozygous affected (IV: 2), normal (IV: 3) and heterozygous parent (III: 1). **d** Multiple alignment of *MLC1* showing complete conservation across different species

In family MR-8, a 7 year old female patient (IV: 2) was the second child to healthy and consanguineous parents from south Punjab in Pakistan (Fig. 3a). The pregnancy was normal and she was delivered at term via caesarian section, with a birth weight of 2.2 kg and head circumference of 41 cm. She developed macrocephaly within the first few months of life, and thereafter showed motor deterioration, and cognitive decline. Her head circumference was 43.5 cm (>95th percentile) at 4 months of age, which indicated macrocephaly. She controlled her head at 8 months of age. She walked independently at 15 months of age, but with some difficulty. She developed recurrent episodes of seizures at 2.5 years of age, as well as mental retardation and progressive motor dysfunction. Brain CT scans performed at 7 years of age, revealed extensive bilaterally symmetrical white matter changes and with subcortical cysts in the bilateral anterior temporal region of the brain (Fig. 3b).

#### Genetic study

WES performed in this patient (IV: 2) is described in methods. In total, 832 variants were selected in the exonic regions and adjacent intronic regions. The variants with allelic frequencies < 0.001% were shortlisted (Additional File 1, Table 4). Variants were validated in the human protein atlas database to check the expression analysis and only one of the rare variants was in the *MLC1* gene (OMIM: 605908) showing higher levels of expression in the brain both at the RNA and protein level. This variant (c.C959A: p.T320K) was not present in (ExAC) and the Genome Aggregation Database (GnomAD) previously. The putative scores of filtered variants were checked and only the *MLC1* gene was predicted as pathogenic. Following *in-silico* tools predicting variant as pathogenic, Mutation Taster (disease_causing), Polyphen2 (Probably damaging), SIFT (Deleterious), GERP and CADD phred (Table 1). After *in-silico* analysis, co-segregation analysis was performed for further validation of the variant and family members showed complete segregation for the *MLC1* variant c.C959A: p.The320Lys (Fig. 3c). Multiple alignment was performed for the *MLC1* gene across orthologous species showing complete conservation in the mutational site (Fig. 3d).

## Discussion

Genetic studies of autosomal recessive ID in consanguineous families can yield variants that segregate with the phenotype of the family and can be considered as disease-causing variants. Our study describes three ID families collected from the South Punjab area of Pakistan. Using combined genotyping, homozygosity-by-descent (HBD) and WES, we identified three novel variants in known genes. In family MR-4, a novel missense variant at nucleotide position c.C605T in exon 8 of the *VPS53* gene, which substitutes the amino acid proline (CCA) with leucine (CTA) (p.Pro202Leu). The variant was confirmed against control samples of the population (*n* = 100) and the *in-silico* predictions of the identified variant were evaluated by using different tools (Table 1). The Golgi associated retrograde protein complex (GARP) consists of four subunits encoded by the *VPS53*, *VPS52*, *VPS54,* and *ANG2* genes. The GARP complex plays an important role to direct retrograde vesicles from endosomes to Trans Golgi network (TGN) [20, 21]. Dysfuctioning of the tethering mechanism between the retrograde vesicles and GARP complex results in an accumulation of lysosomal receptor molecules within the TGN leading to swelling of lysosomes [20, 21]. The previously reported variant, c.A2084; p.Gln695Arg, replaces a glycine with an arginine at the conserved c-terminal domain of the *VPS53* gene. The splice-site variant c.1556 + 5 G > A is predicted to result in a truncated protein with dysfunctioned *VPS53* and GARP complex. Reported work suggests that variants in the *VPS53* subunit may lead to progressive cerebello cerebral atrophy (PCCA) [22]. Phenotypes of our family clinically resemble the PCCA disease. Interestingly, families with PCCA-associated ID from Jewish or Moroccan ancestry were previously reported carrying *VPS53* variants [22]. However, the pathomechanism of *VPS53* variants in PCCA disease is not yet fully understood and future functional studies will be needed.

In family MR-7, a novel homozygous variant was identified in *GLB1* (c.C1318T, p.His440Tyr), where amino acid histidine (CAC) was replaced with amino acid tyrosine (TAC). *GLB1* encodes for an enzyme called beta galactosidase, located in lysosomes which acts as a degrading component for GM1 gangliosides within lysosomes. The accumulation of GM1 gangliosides leads to the lysosomal storage disease due to the deficiency of dissolute of β-galactosidase enzyme [23, 24]. So far, at least 185 disease-causing variants have been reported in the *GLB1* gene [25]. The variant we identified is located within the B-domain 1 of the β-galactosidase structure. B-domain consists of four beta sheets, and variants in the protein core region have been implicated in large structural changes of the β-galactosidase protein structure. These variants could directly impair the lysosomes degrading activity.

In family MR-8, we identified a novel homozygous missense variant in *MLC1* (c.C959A, p.The320Lys), where the amino acid threonine (ACG) is replaced with amino acid lysine (AAG). Variants in the *MLC1* gene have been implicated in megalencephalic leukoencephalopathy with subcortical cysts, an autosomal recessive disorder characterized by macrocephaly, progressive motor and cognitive features, and variable presence of subcortical cysts [26–28]. So far, at least 111 disease-causing variants were identified in the *MLC1* gene [29]. In the present study, we identified a novel (p.The230Lys) variant in *MLC1* segregating with a consistent phenotype in our family. Also, *in-silico* analysis of the identified variant and further co-segregation studies, strongly implicate this variant in the disease of these patients.

## Conclusions

In conclusion, we have been able to identify novel pathogenic variants in the ID-related genes *VPS53*, *GLB1* and *MLC1*. These results allowed us to identify the molecular diagnosis for the recruited families and will also be important for interpreting variants that will be identified in other Pakistani patients and families in the future. The study also helps families to acquire better disease treatment and management.

## Supplementary information

**Additional file 1: Supplementary Table 1.** List of Primers used for Segregation analysis. **Supplementary Table 2.** Exome sequencing Family MR-4 two Patients revealed *VPS53* Mutation. **Supplementary Table 3.** Exome sequencing Family MR-7 two Patients revealed *GLB1* Mutation. **Supplementary Table 4.** Exome sequencing Family MR-8 one Patients revealed *MLC1* gene Mutation.

## Data Availability

The patients’ data are available from the corresponding author on request. The Exome datasets analysed in the study have been deposited in the Harvard dataverse under the following links: 10.7910/DVN/0LN0GK, 10.7910/DVN/CKCSRJ, 10.7910/DVN/PLJLNA. The additional file is also available using the link: 10.7910/DVN/HBDJAP.

## Abbreviations

ID: Intellectual Disability
WES: Whole Exome Sequencing
*VPS53*: Vacuolar Protein Sorting 53
*GLB1*: Galactosidase, beta 1
*MLC1*: Megalencephalic Leukoencephalopathy with Subcortical Cysts
STR: Short tandem repeat
OMIM: Online Mendelian inheritance in men
HBD: Homozygosity-by-descent
CADD: Combined Annotation Dependent Depletion
GERP: Genomic Evolutionary Rate Profiling
ExAC: Exome Aggregation Consortium
GnomAD: Genome Aggregation Database
GATK: The genome analysis toolkit
