# Supplementary material for: Novel variants underlying autosomal recessive intellectual disability in Pakistani consanguineous families
Source: BMC Med Genet. 2020 Mar 24;21:59. doi: 10.1186/s12881-020-00998-z (PMC7092478; doi:10.1186/s12881-020-00998-z)
Supplement: Supplementary file 1 — Additional file 1: Supplementary Table 1. List of Primers used for Segregation analysis. Supplementary Table 2. Exome sequencing Family MR-4 two Patients revealed VPS53 Mutation. Supplementary Table 3. Exome sequencing Family MR-7 two Patients revealed GLB1 Mutation. Supplementary Table 4. Exome sequencing Family MR-8 one Patients revealed MLC1 gene Mutation. [file 12881_2020_998_MOESM1_ESM.docx]

**Title:** Novel variants underlying autosomal recessive intellectual disability in Pakistani consanguineous families

Corresponding Author:

Asif Mir (asif.mir@iiu.edu.pk)

Supplementary Table 1: List of Primers used for Segregation analysis

| Primer Name | Forward Sequence | Reverse Sequence |
| --- | --- | --- |
| *VPS53* | TGTTTCTGGCTTTTCACCTGG | CTTGACAGCACTCATGTTTCAA |
| *GLB1* | CGCTTTCATACATGTCTAGGGT | CTCATCCCCACCCTCACTG |
| *MLC1* | ACTCTGCTCACACCTCCTTC | CCCCACAGGCTTCTCACC |

Supplementary Table 2: Exome sequencing Family MR-4 two Patients revealed *VPS53* Mutation.

| Patient | cDNA | Exon | Protein | Mutation type | Genome AD_All | 1000genome_eas^a^ | ExAC_All | CADD_phred | GERP.._RS | Mutation Taster |
| --- | --- | --- | --- | --- | --- | --- | --- | --- | --- | --- |
| IV:2 | c.C605T | Exon 9 | p.P203L | missense | not found | not found | not found | 10 | 3.83 | Disease causing |
| IV:5 | c.C605T | Exon 9 | p.P203L | missense | not found | not found | not found | 10 | 3.83 | Disease causing |
|  |  |  |  |  |  |  |  |  |  |  |

Supplementary Table 3: Exome sequencing Family MR-7 two Patients revealed *GLB1* Mutation.

| Patient | cDNA | Exon | Protein | Mutation type | Genome AD_All | 1000genome_eas^a^ | ExAC_All | CADD_phred | GERP.._RS | Mutation Taster |
| --- | --- | --- | --- | --- | --- | --- | --- | --- | --- | --- |
| IV:4 | c.C1318T | Exon 13 | p.H440Y | missense | not found | not found | not found | 26 | 5.35 | Disease causing |
| IV:5 | c.C1318T | Exon 13 | p.H440Y | missense | not found | not found | not found | 26 | 5.35 | Disease causing |
|  |  |  |  |  |  |  |  |  |  |  |

Supplementary Table 4: Exome sequencing Family MR-8 one Patients revealed *MLC1* gene Mutation.

| Patient | cDNA | Exon | Protein | Mutation type | Genome AD_SAS | 1000genome_eas^a^ | ExAC_All | CADD_phred | GERP.._RS | Mutation Taster |
| --- | --- | --- | --- | --- | --- | --- | --- | --- | --- | --- |
| IV:1 | c.C959A | Exon 11 | p.T230K | missense | NA | NA | NA | 29.9 | 4 | Disease causing |
|  |  |  |  |  |  |  |  |  |  |  |
